# Comprehensive Analysis of Bacterial Flora in Postoperative Maxillary Cyst Fluid by 16S rRNA Gene and Culture Methods

**DOI:** 10.5402/2012/840483

**Published:** 2012-05-22

**Authors:** Naoto Sano, Yoshio Yamashita, Kazumasa Fukuda, Hatsumi Taniguchi, Masaaki Goto, Hiroshi Miyamoto

**Affiliations:** ^1^Department of Oral and Maxillofacial Surgery, Faculty of Medicine, Saga University, 5-1-1 Nabeshima, Saga 849-8501, Japan; ^2^Division of Microbiology, Department of Pathology and Microbiology, Faculty of Medicine, Saga University, 5-1-1 Nabeshima, Saga 849-8501, Japan; ^3^Department of Microbiology, School of Medicine, University of Occupational and Environmental Health, 1-1 Iseigaoka, Yahatanishi-ku, Kitakyushu 807-8555, Japan

## Abstract

Intracystic fluid was aseptically collected from 11 patients with postoperative maxillary cyst (POMC), and DNA was extracted from the POMC fluid. Bacterial species were identified by sequencing after cloning of approximately 580 bp of the 16S rRNA gene. Identification of pathogenic bacteria was also performed by culture methods. The phylogenetic identity was determined by sequencing 517–596 bp in each of the 1139 16S rRNA gene clones. A total of 1114 clones were classified while the remaining 25 clones were unclassified. A total of 103 bacterial species belonging to 42 genera were identified in POMC fluid samples by 16S rRNA gene analysis. Species of *Prevotella* (91%), *Neisseria* (73%), *Fusobacterium* (73%), *Porphyromonas* (73%), and *Propionibacterium* (73%) were found to be highly prevalent in all patients. *Streptococcus mitis* (64%), *Fusobacterium nucleatum* (55%), *Propionibacterium acnes* (55%), *Staphylococcus capitis* (55%), and *Streptococcus salivarius* (55%) were detected in more than 6 of the 11 patients. The results obtained by the culture method were different from those obtained by 16S rRNA gene analysis, but both approaches may be necessary for the identification of pathogens, especially of bacteria that are difficult to detect by culture methods, and the development of rational treatments for patients with POMC.

## 1. Introduction

Three theories, that is, the retention cyst, cleft cyst, and closed cavity theories, have been proposed for the formation of postoperative maxillary cysts (POMCs) [1–3], which are a long-term delayed complication arising from years to decades after radical operations such as the Caldwell-Luc operation for maxillary sinusitis [4, 5]. This complication is generally known as POMC, although other terms are also found in the literature, such as postoperative buccal cyst, postoperative wangenzyste, mucocele, postoperative paranasal cyst, surgical ciliated cyst, and respiratory implantation cyst [6]. These cysts are considered rare in the West, whereas cysts in the maxillooral region constitute 20% of reported cysts in Japan [6]. They grow painlessly in the maxillary sinus where they are often perceived through swelling or pain in the maxillary mucobuccal fold and buccal region. Other symptoms noted with significant cyst growth include nasal obstruction, rhinorrhea, dysosmia, exophthalmos, and ocular displacement. Bacterial infections in the oral cavity may be closely related to cyst growth and subsequent symptoms [4]. Over 600 bacterial species have been detected in the oral cavity [7], and the majority of these are viable but not yet cultured (VBNC) bacteria [8]. Thus, it is often extremely difficult to identify pathogens in the oral cavity for this type of cyst as well as for other types. Only a few studies have reported the presence of bacteria in the pus of POMCs, but they are unclear [4, 9]. We investigated the bacterial species involved in cyst infection by comprehensively analyzing the bacterial flora in POMC fluid by 16S rRNA gene analysis.

## 2. Materials and Methods

### 2.1. Subject Population and Characteristics

 Study patients included 7 males and 4 females with POMC (mean age: 58.8 years; range: 33–87 years). All patients had a history of surgery for sinusitis (14–50 years prior; mean: 34.6 years prior). Cyst fluid was obtained by aspiration, and most fluid was dark brown in color. The fluid was serous in 4 patients and viscous in 7 patients. In all patients, signs of inflammation were evident in the affected region, such as swelling, redness, pain, fever, and/or nasal obstruction (Table 1). Antibiotics were administered prior to the collection of cyst fluid in 9 patients, but it was unclear in 1 patient.

### 2.2. Collection of Samples

 After gargling with gluconic acid, chlorhexidine (ConCool F; WelTec, Japan), the most swollen maxillary mucobuccal area was disinfected with povidone-iodine solution before direct intracystic puncture using an 18-G needle (Terumo Corporation, Tokyo, Japan) and aspiration. Samples were immediately transferred into sterile tubes containing CO_2_ (Kenkiporter; Terumo Clinical Supply Co., Ltd., Tokyo, Japan) to ensure the preservation of anaerobes. One tube was used for the identification of pathogens by bacterial culture testing, while the other was subjected to DNA extraction within 1 h of collection.

### 2.3. DNA Extraction Method

Sample fluid was combined with an equal volume of sterile distilled water and DNA extracted using a GFX Genomic Blood DNA Purification Kit (GE Healthcare UK Limited, Little Chalfont, UK) according to the manufacturer's instructions.

### 2.4. Identification of Microbiota

The primers E341F (5′-CCTACGGGAGGCAGCAG-3′) and E907R (5′-CCGTCAATTCMTTTRAG-3′) [10] were used to amplify the 16S rRNA gene. The primers were assessed using the Probe Match program on the Ribosomal Database Project II website (RDP-II, http://rdp.cme.msu.edu/). The high coverage ratios of E341F and E907R were 93.1% and 96.1%, respectively, for 138,807 bacterial 16S rRNA genes.

### 2.5. PCR Conditions

The 16S rRNA gene was amplified from chromosomal DNA extracted from cyst fluid using the GFX Genomic Blood DNA Purification Kit (GE Healthcare UK Limited) for clone library construction. All PCR amplification mixtures (25 *μ*L) contained 1 × GeneAmp PCR buffer (10 mM tris-HCl, pH 8.3; 50 mM KCl; 1.5 mM MgCl_2_; 0.01% wt/vol gelatin), 0.2 mM of each deoxynucleoside triphosphate, 2.5 pmol of each primer, 1.25 U of AmpliTaq Gold DNA polymerase (Applied Biosystems, Foster City, CA, USA), and 1 *μ*L of the template DNA solution. The reaction mixtures were incubated in a thermocycler at 96°C for 5 min, followed by 27 cycles at 96°C for 30 s, 55°C for 30 s, and 72°C for 1 min, with a final extension at 72°C for 7 min. The PCR products (approximately 580 bp) were determined by gel electrophoresis using a 2.0% agarose gel. After confirming the product size, the product was purified and added to 25 *μ*L of TE buffer using a Centricon YM-100 concentrator (Millipore, Bedford, MA, USA).

### 2.6. Clone Library Construction and Nucleotide Sequence Determination [10]

 PCR products were cloned using a TOPO TA Cloning kit (Invitrogen, Carlsbad, CA, USA) according to the manufacturer's instructions. Transformation was performed using competent *E. coli* TOP10 cells provided by the manufacturer. A total of 96–288 white colonies were randomly selected from each clone library for sequencing analysis. To prepare a template for sequencing analysis, a partial fragment was amplified from the cloning vector (pCR II) containing an inserted PCR product using M13 forward, M13 reverse, and AmpliTaq Gold DNA polymerase. The reaction mixtures were incubated in a thermocycler at 95°C for 2 min, followed by 20 cycles at 95°C for 30 s, 60°C for 30 s, and 72°C for 30 s, with a final extension at 72°C for 3 min. After the primers and dNTP were eliminated from the PCR mixture using an ExoSAP-IT Kit (USB, Cleveland, OH, USA) according to the manufacturer's instructions, a 1 *μ*L aliquot was used as a template for the sequencing reaction. The sequencing reactions used the M13 reverse primer and a BigDye Terminator Cycle Sequencing kit v3.1 (Applied Biosystems, Carlsbad, CA, USA). The nucleic acid sequences were determined using a 3130xl Genetic Analyzer (Applied Biosystems). Homology searches were performed using the basic local alignment search tool (BLAST) with an in-house software system and database, which contained type strains (5,878 species) from the RDP-II website (http://rdp.cme.msu.edu/) and DNA Data Bank of Japan (http://www.ddbj.nig.ac.jp/). A level of 98% sequence identity was used as the cutoff for the identification of a species taxon. Phylogenetic trees were constructed using the neighbor-joining method of Saitou and Nei [11].

### 2.7. Bacterial Culture

 Pathogens were isolated from intracystic fluid at the central and clinical laboratories in Saga University Hospital. Intracystic fluid from each patient was inoculated onto 6 different types of agar plates, that is, sheep blood agar, sheep blood agar supplemented with phenylethyl alcohol, bromothymol blue agar, chocolate agar, prereduced *Brucella* HK (RS) agar, and prereduced *Brucella *HK-PV agar (Kyokuto Pharmaceutical Industrial Co., Ltd., Tokyo, Japan). The plates were cultured aerobically or anaerobically at 37°C for 2–7 days. Based on the abundance and morphology of colonies, 2–5 colonies per sample were isolated individually on the agar plates, as described above, and reincubated aerobically or anaerobically at 37°C for 2–7 days. The isolates were identified using VITEK2 (Biomerieux Japan Co., Ltd., Tokyo, Japan), WalkAway (Siemens Healthcare Diagnostics K.K., Tokyo, Japan), or the RapID-ANA II system (Remel Inc., Lenexa, KS, USA) according to the manufacturer's instructions.

## 3. Results

**Figure 1:**
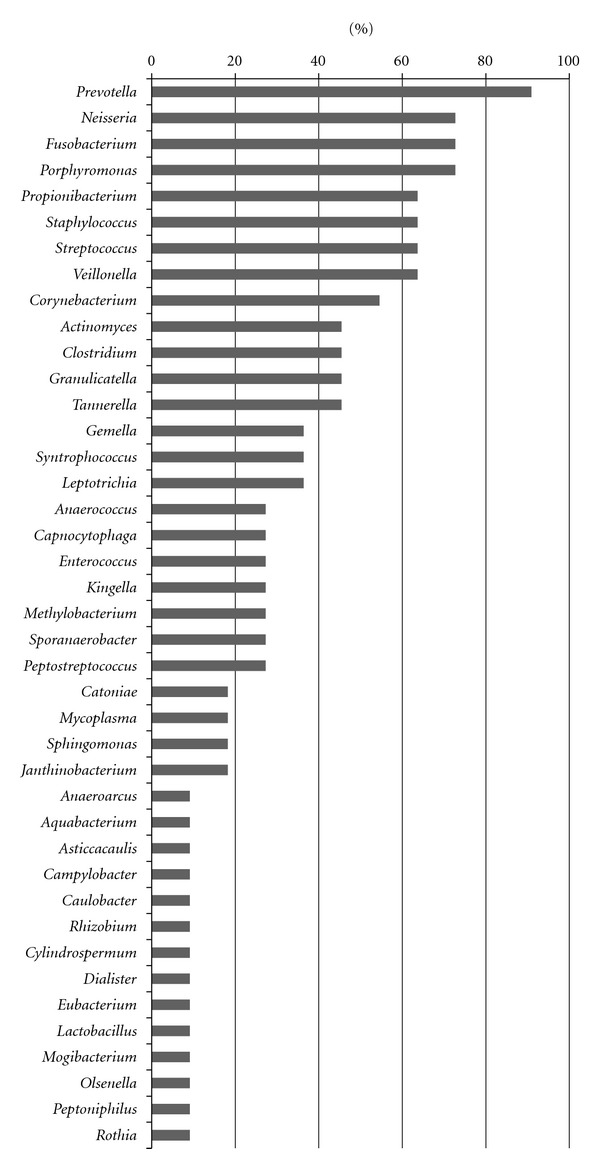
Prevalence (%) of bacterial genera detected in postoperative maxillary cyst fluids from 11 patients by 16S rRNA gene analysis. The identity of 1114 clones was determined by sequence analysis.

**Figure 2:**
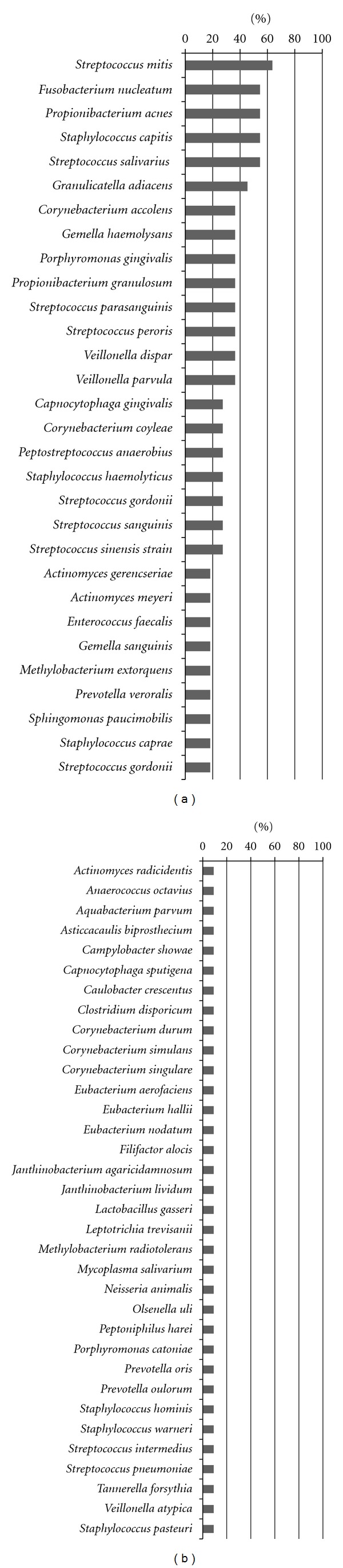
Prevalence (%) of bacterial species detected in postoperative maxillary cyst fluids from 11 patients by 16S rRNA gene analysis. A total of 1114 clones were classified. The identity of 1114 clones was determined by sequence analysis.

**Figure 3:**
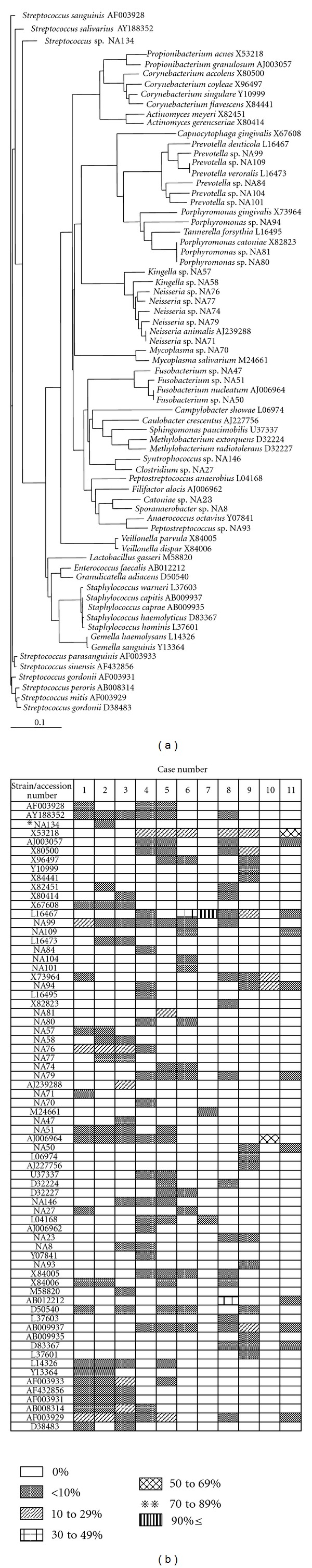
(a) Phylogenetic tree of bacterial phylotypes detected in patients with postoperative maxillary cysts. The marker bar represents a 10% difference in the nucleotide sequence. (b) Each column of boxes represents the bacterial profile/patients. grey-, stripe-, square-, slant square-, and vertically stripe-shaded boxes indicate the presence of species detected at <10%, 10–29%, 30–49%, 50–69%, and >90% of the total number of clones analyzed, respectively. Clear boxes indicate that species were not detected (below the limit of detection). *NA indicates the name of a strain (clone), while other codes such as AF, AY, and X are the accession numbers of the type strains of species. The order of strain/accession numbers (b) corresponds to the order of the phylogenetic tree (a). **None.

The phylogenetic identity of 1139 16S rRNA gene clones was determined by sequencing 517–596 bp for each clone. A total of 1114 clones were classified while the remaining 25 clones were unclassified (Table 2). The 25 clones showed less than 85% homology to nucleotide sequences present in the database. Overall, 41 genera and 64 species were detected. The genera and species are listed in Figures 1 and 2, respectively, in the order of decreasing prevalence. *Prevotella* (91%), *Neisseria *(73%), *Fusobacterium* (73%), and *Porphyromonas* (73%) had the highest prevalence in all patients (Figure 1). *S. mitis* (64%), *Fusobacterium nucleatum* (55%), *P. acnes* (55%), *S. capitis* (55%), and *S. salivarius* (55%) were detected in more than 6 of the 11 patients (Figure 2).  Figure 3(a) shows 70 of the most prominent taxa among the 105 taxa/species detected. The taxa detected fell into 5 bacterial phyla, that is, *Firmicutes, Fusobacteria, Actinobacteria, Proteobacteria*, and *Bacteroidetes*. *Spirochaetes* such as *Treponema* spp. were not detected in any of the samples by 16S rRNA gene analysis. Differences in the bacterial profiles of each patient are shown in Figure 3(b) and Table 2. The mean numbers of taxa/species detected in purulent and serous fluids were 28 (range: 13–41) and 12 (range: 3–23), respectively.

As shown in Table 2, 5 of the 11 samples were negative for bacterial growth, regardless of whether inflammation was observed. Four of the five negative samples were collected from patients who had received antibiotics. The mean numbers of taxa/species detected by 16S rRNA gene analysis in POMC fluids of patients who had received macrolides and *β*-lactams were 27 (range: 6–39) and 23 (range: 17–29), respectively (Tables 1 and 2). Although *S. sanguinis* was identified in case 3 by culture method, this species was not detected by 16S rRNA gene analysis. *Klebsiella pneumoniae*, *Streptococcus. anginosus*, *Peptoniphilus asaccharolyticus*, and *Finegoldia magna* were identified in case 5, but they were not detected by 16S rRNA gene analysis. *Prevotella melaninogenica *is not the same sample as *P. denticola* even by biochemical methods, although both are *Prevotella* species. Streptococci *viridans* were also identified by the culture method for cases 10 and 11, but they were not detected by 16S rRNA gene analysis. Only 3 taxa/species were found in case 10 who had received no antibiotics prior to sample collection, that is, *F. nucleatum* (54%), *Porphyromonas gingivalis* (29%), and *Porphyromonas* spp. (17%) (Table 2). In case 8, *Enterococcus faecalis* was the most prevalent bacteria detected by both the culture method and 16S rRNA gene analysis, comprising 35% of the total population (Table 2). Only in case 8, the species identified by the culture method matched that detected as the most prevalent bacteria species by 16S rRNA gene analysis.

## 4. Discussion

Although more than 600 species of bacteria are believed to be present in the oral cavity [7], the majority of these bacteria are VBNC [8], that is, bacteria not yet detected by currently available culture methods. Therefore, we conducted a comprehensive survey of the bacterial flora in POMC fluid by 16S rRNA gene analysis and culture methods to better determine the actual abundance of bacteria, including VBNC bacteria.

Infections originating in the oral cavity are complex because of the presence of a large numbers of bacterial species [12, 13], which makes it difficult to determine the bacteria involved in most oral infections. As previously stated, there are 3 current theories regarding the formation of POMCs, and the relationship between the formation of POMCs, their progression, and bacteria is not well understood. Very few studies have identified the bacteria involved in POMC infections [4, 9]. Bacterial infections may be closely linked to cyst growth and the progression of its symptoms because the maxillary sinus floor is adjacent to the oral cavity, and bacteria reach the cyst via the teeth and periodontal pockets despite the cyst cavity being isolated by the cyst wall. In this study, we aseptically collected intracystic POMC fluid and compared the accuracy of identification of bacteria by conventional culture methods and 16S rRNA gene analysis.

As shown in Figure 3(a), a high bacterial diversity was observed in the POMC fluid, and the bacterial flora in this fluid is shown for each patient (Figure 3(b), Table 2). All POMC fluids contained bacteria, and purulent fluids contained higher numbers of bacteria species/taxa than serous fluids (Tables 1 and 2), suggesting that oral bacterial infections of POMC may be involved in inflammatory symptoms, such as cheek swelling and pain caused by POMC, and symptom progression. *Prevotella*, *Neisseria*, *Fusobacterium*, *Porphyromonas*, and *Propionibacterium* were detected in more than 70% of all patients (Figure 1), while *S. mitis*, *F. nucleatum, P. acnes*, *S. capitis*, and *S. salivarius* were detected in more than 50% of all patients (Figure 2). *P. acnes *was the dominant bacterium in 6 of 11 patients (Figure 3(b)). It is present on the skin, in the oral cavity, and the intestinal mucosa where it is known to be the causative agent of a wide range of diseases and opportunistic infections, including alveolar abscesses, maxillary sinusitis, osteomyelitis, meningitis, noma, pericarditis, sepsis, hepatitis, granuloma, acne, and orbital abscesses [14–16]. *S. mitis*, which was detected in 7 patients (Figure 3(b)), is an indigenous oral bacterium that is primarily present in the buccal mucosa and on tooth surfaces. It is a potential causative agent of endocarditis [17, 18]. *P. denticola*, which was detected in 6 patients (Figure 3(b)), is an indigenous oral bacterium and commonly detected in subgingival biofilm of periodontitis patients [19], *F. nucleatum*, which was detected in 6 patients (Figure 3(b)), is associated with periodontitis, and according to Kuriyama et al. [20, 21] it is often present in an abscess along with the *anginosus* group of *Streptococci*. Other bacterial species were detected, including *E. faecalis*, *S. capitis*, *P. gingivalis*, *S. haemolyticus*, *S. parasanguinis*, *S. salivarius*, and *S. sinensis*, but they were present at a lesser extent in multiple cases compared with the species mentioned above. *E. faecalis* was the dominant bacteria in case 8 (Table 2), and it is normally part of the indigenous bacterial flora in the human intestinal tract, although it can cause urinary tract infections, endocarditis, meningitis, and opportunistic infections [22]. *E. faecalis* is often found in the teeth [23] during root canal treatment, where it is associated more with the asymptomatic initial period of root canal bacterial infections than with symptomatic root canal bacterial infections. It is significantly more common in cases of failed root canal treatment than of early root canal bacterial infections [24].

There are very few reports on the potential origin of the microbiota in POMC fluid. In case 8, the potential origin might be an endodontic source because only *E. faecalis* was detected by both the culture method and 16S rRNA gene analysis. Case 10 received no antibiotics, and *F. nucleatum* and *P. gingivalis*, which are closely related to periodontitis, were detected by 16S rRNA gene analysis, suggesting that the origin might be a periodontal source. However, *Treponema* spp. are often detected in periodontal diseases, and none were found in any of the samples. Further large-scale studies are required to understand the potential origin of the microbiota in POMC fluid.

 Only in case 8 did the most dominant bacterial species identified by 16S rRNA analysis matched that identified by the culture method. In general, it is difficult and time consuming to determine the causative agents of odontogenic infections by culture methods because more than 10 types of very small-sized colonies, including normal bacterial flora, are often observed during the first culture examination of oral clinical samples. Several colonies are selected and used for subsequent species identification tests. The results obtained by the culture method were different from those obtained by 16S rRNA gene analysis. There are several possible reasons for this result. It is most probable that the number of bacteria identified by conventional culture methods differs from the actual number present because the actual causative agents might not be detected in a culture medium as a result of their VBNC status or competition with other bacterial species for survival, thereby leading to a negative result. Long-term clinical administration of macrolide antibiotics is often followed in patients with maxillary sinusitis [25]. Macrolide antibiotics had been administered by general practitioners prior to definite POMC diagnosis in 6 of the 9 patients in our study. Thus, it is possible that some bacteria were eliminated by the antibiotics used. In addition, DNA extraction is not limited solely to living individuals because all environmentally present 16S rRNA genes are amplified by universal primers in community structure analysis. Therefore, it is possible that the remnants of DNA of dead bacterial cells were also amplified. We could not identify the possible causative bacteria or the susceptibilities of antimicrobial agents to the possible causative bacteria by 16S rRNA gene analysis; therefore, the conventional culture method was necessary to identify species and develop rational treatments for patients with odontogenic infections. In addition, odontogenic infections are often mixed, and the true pathogen may not be the dominant organisms in the pus where it may be hidden by larger numbers of opportunistic organisms. The numbers of low-prevalence taxa/species are shown in Table 2 as other spp. In addition, some of the low-prevalence taxa/species are also shown in Figures 1 and 2. However, the significance of the low-prevalence taxa/species for ecology or etiology remains unclear in this study.

## 5. Conclusions

 We identified the bacterial flora present in POMC fluid by 16S rRNA gene analysis and conventional culture methods. Bacterial fell into 41 genera and 64 species were detected in the 11 patients with POMC bacterial infections, which may be closely involved in both inflammatory symptoms, such as cheek swelling and pain caused by POMC, and symptom progression. The results obtained by the culture method were different from those obtained by 16S rRNA gene analysis, but both approaches may be necessary for the identification of pathogens, especially of bacteria that are difficult to detect by culture methods, and the development of rational treatments for patients with POMC. Further large-scale studies are required to understand the potential origin of the microbiota in POMC fluid.

## Figures and Tables

**Table 1:** Characteristics of postoperative maxillary cyst patients.

Case no.	Age	Sex	Previous operation (y)	Side of POMC	Aspect of pus	Antibiotics	Inflammatory symptom				
							Pain	Redness	Swelling	Fever	Nasal obstruction
1	60	M	1981	L	Brown, purulent	Macrolide (CAM)	+	+	+	−	−
2	67	F	1971	L	Yellow, serous	Cephem (CFDN)	+	+	+	−	−
3	59	M	1971	L	Dark brown, purulent	Penicillin (AMPC/CVA)	+	+	+	−	−
4	50	F	1978	R	Dark brown, purulent	Macrolide (CAM)	+	+	+	+	+
5	60	M	1971	R	Dark brown, purulent	Macrolide (CAM)	+	+	+	−	−
6	37	F	1991	L	Dark brown, purulent	Macrolide (CAM)	+	+	+	−	−
7	54	F	1971	R	Dark brown, serous	Macrolide (CAM)	+	+	+	−	+
8	71	M	1977	L	Dark brown, purulent	Macrolide (CAM)	+	+	+	+	+
9	87	M	1971	L	Dark brown, serous	Cephem (CFPN-PI)	+	+	+	−	−
10	69	M	1961	R	Dark brown, serous	—	+	+	+	−	+
11	33	M	1997	R	Yellowish white, purulent	Unknown	+	+	+	−	−

POMC, postoperative maxillary cyst; y, years; L, left; R, right; M, male; F, female CAM, clarithromycin; CFDN, cephalosporin; AMPC/CVA, amoxicillin-potassium clavulanate combination, CFPN-PI, cefcapene pivoxil.

**Table 2:** Results of culture and 16S rRNA gene analyses in postoperative maxillary cyst fluids from 11 patients.

Case no.	Culture method	Dominant taxa detected by 16S rRNA gene analysis** (%)						No. of total spp. detected	No. of clones	
		1st	2nd	3rd	4th	5th	***No. of other spp.		classified	unclassified
1	Negative	*Neisseria* spp. 24%	*Streptococcus mitis* 15%	*Prevotella spp.*10%	*Streptococcus parasanguinis *5%		35 46%	39	206	22
2	Negative	*Streptococcus mitis* 21%	*Neisseria spp.* 19%	*Streptococcus parasanguinis* 9%	*Streptococcus sinensis* 8%	*Streptococcus salivarius *7%	18 40%	23	101	2
3	*Streptococcus* *sanguinis *	*Streptococcus parasanguis* 15%	*Streptococcus peroris* 12%	*Neisseria spp.* 12%	*Neisseria animalis* 10%	*Streptococcus sinensis* 8%	24 43%	29	100	0
4	Negative	*Propionibacterium acnes* 19%	*Porphyromonas spp.* 11%	*Peptostreptococcus anaerobius* 9%	*Streptococcus mitis* 5%		32 56%	36	119	0
5	*Klebsiella* *pneumoniae Streptococcus* *anginosus Peptoniphilus* *asaccharolyticus Finegoldi magna *	*Propionibacterium acnes *14%	*Streptococcus mitis* 12%	*Porphyromonas catoniae* 10%	*Sphingomonas paucimobilis* 7%	*Prevotella spp. *6%* Neisseria spp. *6%* Fusobacterium nucleatum *6%	36 40%	41	126	0
6	*Streptococcus viridans Parvimona micros Prevotella* *melaninogenica *	*Prevotella denticola* 35%	*Propionibacterium acnes* 21%				15 44%	17	75	0
7	Negative	*Prevotella denticola* 92%					5 8%	6	62	0
8	*Enterococcu faecalis*	*Enterococcus faecalis* 35%	*Propionibacterium acnes* 22%	*Neisseria spp.* 6%			19 37%	22	86	1
9	Negative	*Propionibacterium acnes* 25%	*Staphylococcus capitis* 20%	*Prevotella denticola* 12%	*Corynebacterium accolens* 12%	*Staphylococcus haemolyticus* 7%	12 26%	17	61	0
10	*Streptococcus viridans	*Fusobacterium nucleatum* 54%	*Porphyromonas gingivalis* 29%	*Porphyromonas spp.* 17%			0%	3	93	0
11	*Streptococcus viridans*	*Propionibacterium acnes* 61%	*Staphylococcus haemolyticus* 8%	*Prevotella denticola* 8%	*Fusobacterium nucleatum* 7%	*Porphyromonas spp.* 6%	8 9%	13	85	0

									1114	25

*Streptococcus viridans comprehends a group composed of *Streptococcus sanguis* group, *Streptococcus milleri* group, and *S. mitis*. Species of Streptococus *viridans* are not determined in this study.

**(%) the percentage of bacteria indicates the percentage of bacteria detected versus the total bacterial population.

***Bacteria constituting less than 5% of the total bacterial population are grouped together as other spp.
